# Technology for 3D System Integration for Flexible Wireless Biomedical Applications

**DOI:** 10.3390/mi9050213

**Published:** 2018-05-02

**Authors:** Wen-Cheng Kuo, Chih-Wei Huang

**Affiliations:** Department of Mechanical and Automation Engineering, National Kaohsiung University of Science and Technology, 2 Jhuoyue Rd., Nanzih, Kaohsiung 811, Taiwan; u0514803@nkfust.edu.tw

**Keywords:** parylene, 3D package, biomedical system, MEMS

## Abstract

This paper presents a new 3D bottom-up packing technology for integrating a chip, an induction coil, and interconnections for flexible wireless biomedical applications. Parylene was used as a flexible substrate for the bottom-up embedding of the chip, insulation layer, interconnection, and inductors to form a flexible wireless biomedical microsystem. The system can be implanted on or inside the human body. A 50-μm gold foil deposited through laser micromachining by using a picosecond laser was used as an inductor to yield a higher quality factor than that yielded by thickness-increasing methods such as the fold-and-bond method or thick-metal electroplating method at the operation frequency of 1 MHz. For system integration, parylene was used as a flexible substrate, and the contact pads and connections between the coil and chip were generated using gold deposition. The advantage of the proposed process can integrate the chip and coil vertically to generate a single biocompatible system in order to reduce required area. The proposed system entails the use of 3D integrated circuit packaging concepts to integrate the chip and coil. The results validated the feasibility of this technology.

## 1. Introduction

A growing trend is observed in the minimization of device size and the integration of various components into a single device. The chip-embedded flexible packaging technology is required to obtain small, rapid, and reliable devices. Flexible packaging entails vertically integrating the active and passive components on a flexible substrate to reduce the required area.

Previously, pieces of medical inspection equipment were considerably large and could not appropriately detect diseases without being in contact with human bodies. An implantable wireless bio-microsystem was then developed to satisfy medical requirements. Schnakenberg et al. [1] proposed an intravascular pressure monitoring system with parylene-C coating for monitoring blood pressure in hypertensive diseases or pulse rate in arrhythmia. Coosemans et al. [2] proposed an autonomous bladder pressure monitoring system, which registers and wirelessly transmits the bladder pressure to the doctor. Li et al. [3] proposed an integrated flexible ocular coil for power and data transfer in retinal prostheses. A parylene-based device with a gold coil could be directly integrated with multielectrode arrays and parylene-based packages incorporating application-specific integrated circuits (ASICs).

High-performance, high-density, and compact electronic products have been developed not only for portable electronic devices [4] but also for biomedical systems. In the 1990s, a system-on-chip (SoC) [5] was developed for integrating all components on a monolithic substrate to enhance the overall speed of a system and reduce power consumption. Yang et al. [6] presented a PDMS-based package with an implantable complementary metal-oxide-semiconductor (CMOS) drug delivery SoC, in which a wireless controller/actuation circuitry and a drug delivery array are monolithically integrated. Chiu et al. [7] proposed a pain control system with a flexible substrate, integrating a flat spiral induction coil and CMOS-based SoC. However, system efficiency is limited by the inadequate performance of a radio frequency (RF) circuit and decoupling capacitors due to size constrains. To overcome this limitation, Chang et al. [8] proposed a three-coil wireless power transfer system using a 3D package. However, the integration of chips and passive components in high-density electronic devices also satisfies both size and performance requirements. The 3D package [9] was used to satisfy both requirements.

Initially, system-in-package (SiP) technology was used to develop 3D packaging by using wire bonding [10] and a solder ball [11], integrating all the components along the Z-axis. For example, Chandler [12] integrated an optoelectronic and radio subsystem by using a 3D SiP process, which reduced the weight, area, and cost of the system. Yole Inc. [13] proposed a wafer-level packaging/3D IC through silicon via by using deep reactive ion etching (DRIE) or laser drilling to create the via and interconnection techniques and reduce the interconnection length in order to improve the performance. This technique could integrate different chips into a system, including RF, logic circuit, and micro-electro-mechanical system (MEMS) devices. Solid materials, such as silicon and printed circuit board (PCB), were used as substrates. Berenyi et al. [14] used polyimide (PI) as a substrate and then employed ultraviolet laser cutting and integrated five IC chips on the flexible PI substrate for reducing the area by 55% without causing the overheating problem. In this study, interconnections were imbedded with microvoids by using the 3D IC process, and the chip and coil were integrated using the SiP process to reduce the area.

The bio-implantable systems were used in batteryless implantable CMOS SoC [7] and retinal prostheses [3]. A wireless bio-implantable system should be biocompatible, which engenders a problem. The materials to be used must be nontoxic, nonthrombogenic, noncarcinogenic, and nonirritant [15]. Gold and parylene are biocompatible materials of United States Pharmacopeia (USP)-VI grade, which can be used for implantable systems [16]. In such systems, parylene can serve as a flexible substrate to form conformal coatings for gold-based interconnections and chips to integrate the chips and induction coils into a system. In this work, a silicon substrate was used as a mold to create a trench for chip embedding and deposit/pattern gold as an interconnection between conduction pads, and a coil was then pasted on the interconnection. Parylene deposited between the coil and interconnections was considered as an insulation layer and capsulation. When the capsulation was completed, the silicon substrate could be removed from the system after dicing.

## 2. Design

### 2.1. Theory of Induction Coils

An induction coil is used for energy and signal communication inside a wireless microsystem. The Q-factor is used for determining quality [17], which can be calculated as follows:

(1) $$Q=\frac{\omega L_{s}}{R_{s}}\left[1-{\left(\frac{\omega }{\omega_{r}}\right)}^{2}\right]$$

where *L_s_* and *R_s_* are the inductance and resistance, respectively. *ω* and *ω_r_* are the operating and resonant frequencies of the inductor, respectively. Based on (1), the inductor must be operated below self-resonance to avoid the minimization of the Q-factor incurred by the loss of self-resonance.

O’Driscoll [18], Sauer [19], and Vaillancour [20] have revealed that the energy loss decreased for a frequency ranging from 1 to 10 MHz. Flexible inductors are commonly used in implantable biomedical devices to wirelessly transmit energy and data. However, the device size and operational frequency (approximately 1 MHz) [7] used for energy transmission limits the quality factor (Q-factor) when coupling with external circuits [21].

In this paper, 50 μm of gold was used by micro-laser machining to enhanced the Q-factor at 1 MHz because of the skin effect, thereby reducing the corresponding resistance of the inductor [22] to replace the time consuming fold-and-bond [23] and thick-metal electroplating methods [24].

### 2.2. Dummy Chip Experiment

**Figure 1:**
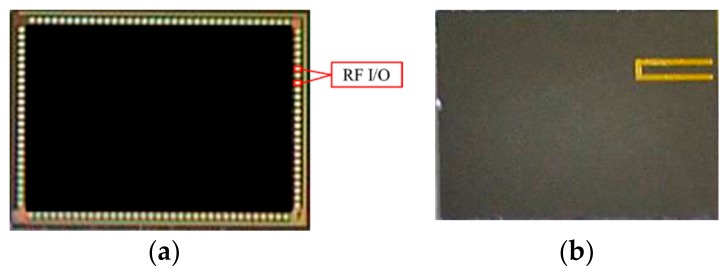
Contact pad position for actual energy-harvesting chip (**a**) and dummy chip (**b**).

This study integrated a high-Q inductor and an energy-harvesting chip into a wireless microsystem. The system required an SoC to integrate the logic components into a chip. To make the process feasible, a dummy chip with the same size as an energy-harvesting chip (4 mm × 3 mm) was used. Moreover, the same RF input/output pads (80 μm × 80 μm, spacing 21 μm) were used. In the designed dummy chip, for electricity conduction throughout the system, the two RF I/O pads were one interval between adjacent connected using gold interconnection, as shown in Figure 1. The height of the dummy chip is 250 μm without thinning process.

### 2.3. 3D Packaging Process Design

**Figure 2:**
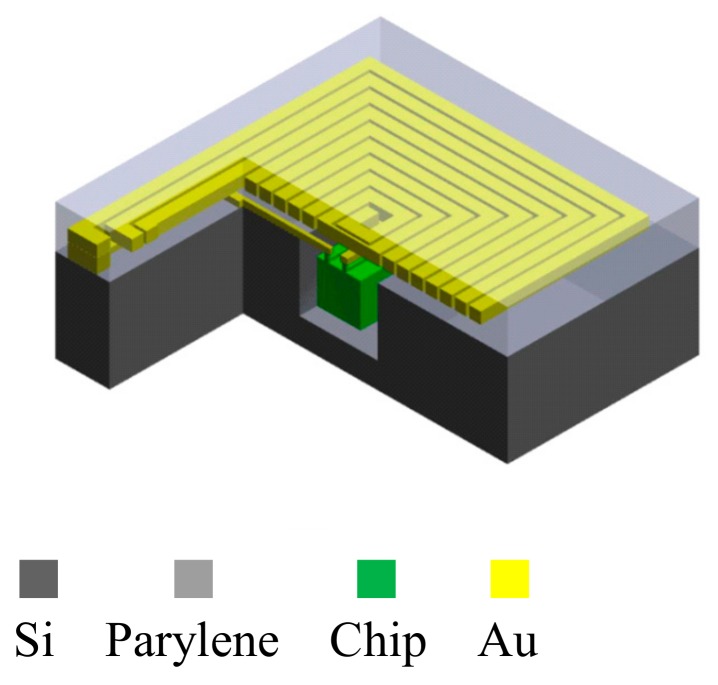
Cross-section of the wireless biomedical microsystem.

This study proposes a new 3D bottom-up packing technology for chips, induction coils, and interconnections. Parylene was used as the flexible substrate for bottom-up embedding of the chip, insulation layer, interconnection, and inductors to create a flexible wireless biomedical microsystem. A silicon wafer was used to integrate the chip and parylene substrate. The trench was etched using inductively coupled plasma (ICP)-DRIE to fit the chip and parylene. ICP-DRIE could be used to etch the precise depth required for the chip and parylene substrate. The dump chip, combined with the aligned key in the silicon wafer, can fit the trench by aligning the X-, Y-, and Z-axes. A parylene insulation layer was deposited between the metal connections for insulation, and reactive ion etching (RIE) was used for opening the parylene insulation layer under the connection pad. Gold was then deposited inside the opening to create vias between metal layers. The design concept is presented in Figure 2.

## 3. Fabrication

### 3.1. Induction Coil Fabrication

**Figure 3:**
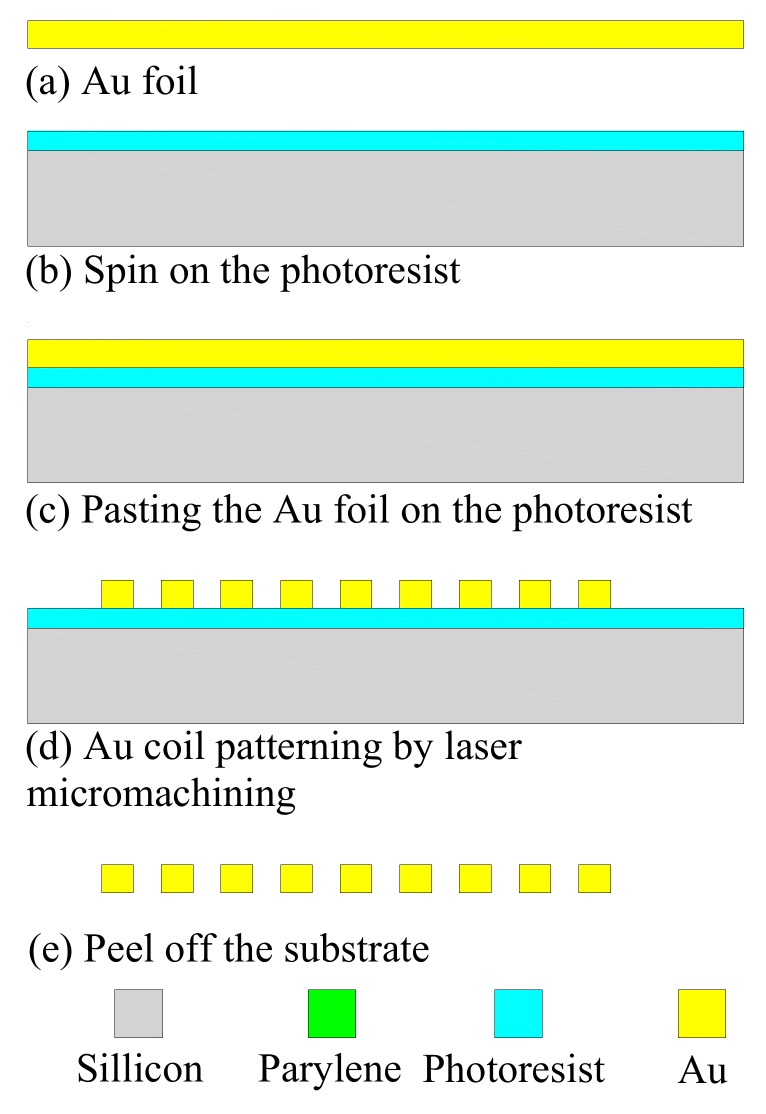
Process of coil fabrication through laser micromachining.

The induction coil was fabricated with the technology used in [22]. Parylene-C was used as the polymer substrate, and the coil was patterned using a pulsed laser, as shown in Figure 3. Compared with the fold-and-bond [23] and thick-metal electroplating [24] process, the proposed process can easily and rapidly generate an induction coil with a larger thickness and higher quality factor. The proposed process can be used for fabricating an implantable bio-microsystem for energy and signal communication.

### 3.2. Induction Coil and Chip Integration

**Figure 4:**
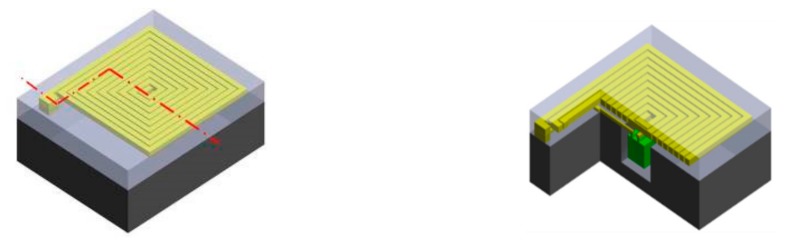

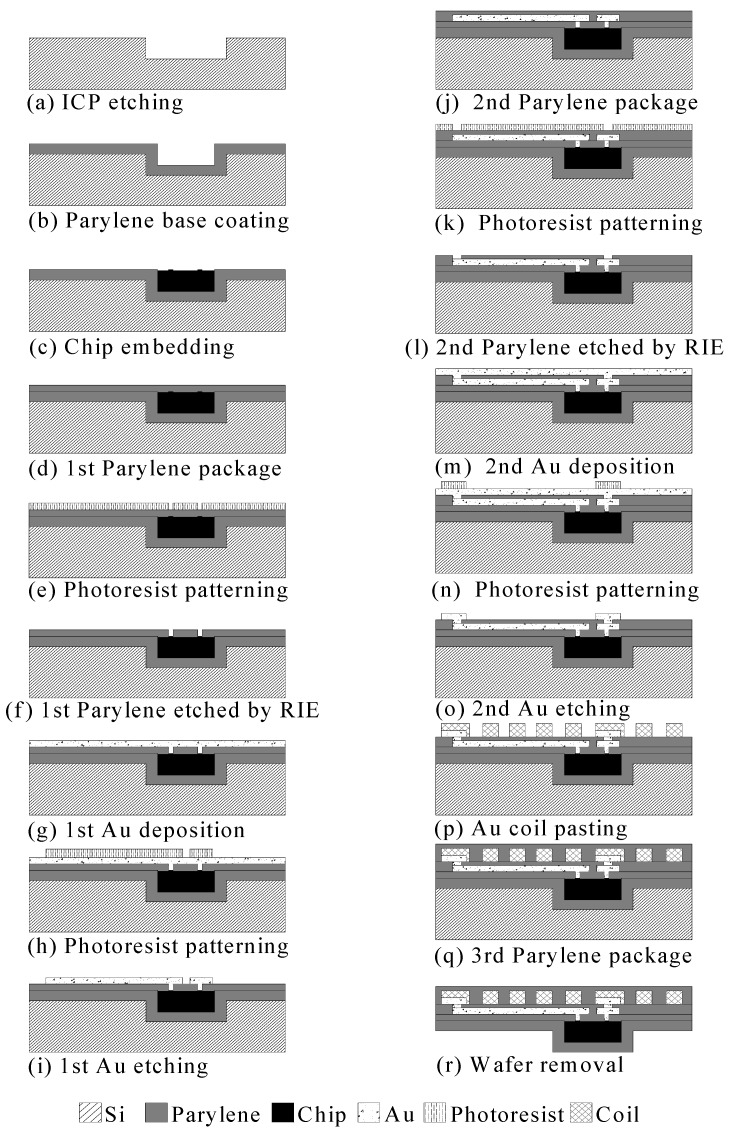
Fabrication process for system integration.

The 3D bottom-up integration process of the chip is shown in Figure 4. The process is as follows:

Step a: ICP etching

Photoresist AZ4620 with a thickness of approximately 15 µm was spun on a silicon wafer as an etching mask, and ICP silicon etching was used for deep silicon etching (250 µm).

Step b: Parylene coating

30 µm of parylene was deposited on the wafer as a flexible substrate.

Step c: Chip embedding

The sample was heated up to 80 °C and the chip was embedded.

Step d: First parylene package

After the chip was embedded, parylene measuring 2 µm was deposited as the first isolation layer.

Step e: Photoresist patterning

Photoresist AZ4620 of approximately 5 µm thickness was spun on the parylene base as the etching mask, followed by a pattern transfer process. Concerning the thickness variation of parylene before and after baking, the temperature of soft and hard bake is 80 °C, which is smaller and close to the glass transition point (80–100 °C) of parylene C, and the linear coefficient of expansion is about 3.8 × 10^−^^5^/°C. The thickness variation before and after baking is small.

Step f: First parylene etched using RIE

Parylene was etched using RIE. The pad on the chip was then exposed for via patterning. The AZ4620 will be removed after etching.

Step g: First gold deposition

Gold (2000 Å) was deposited using an electron beam evaporator as the interconnection layer.

Step h: Photoresist patterning

Photoresist S1818 of approximately 2 µm thickness was spun on the gold base as the etching mask for via patterning, followed by a pattern transfer process.

Step i: First gold etching

Gold wet etching was used for the etchant solution, and gold was patterned for the conductive wire.

Step j: Second parylene package

After gold etching, parylene measuring 2 µm was deposited as the second isolation layer.

Step k: Photoresist patterning

Photoresist AZ4620 of approximately 5 µm thickness was spun on the parylene base as the etching mask, followed by a pattern transfer process.

Step l: Second parylene etched by RIE

Parylene was etched using RIE. The parylene hole was then used for depositing the interconnection metal.

Step m: Second gold deposition

Gold (2000 Å) was again deposited using the E-beam evaporator as the interconnection layer.

Step n: Photoresist patterning

Photoresist S1818 of approximately 2 µm thickness was spun on the second gold base as the etching mask, followed by a pattern transfer process.

Step o: Second gold etching

**Figure 5:**
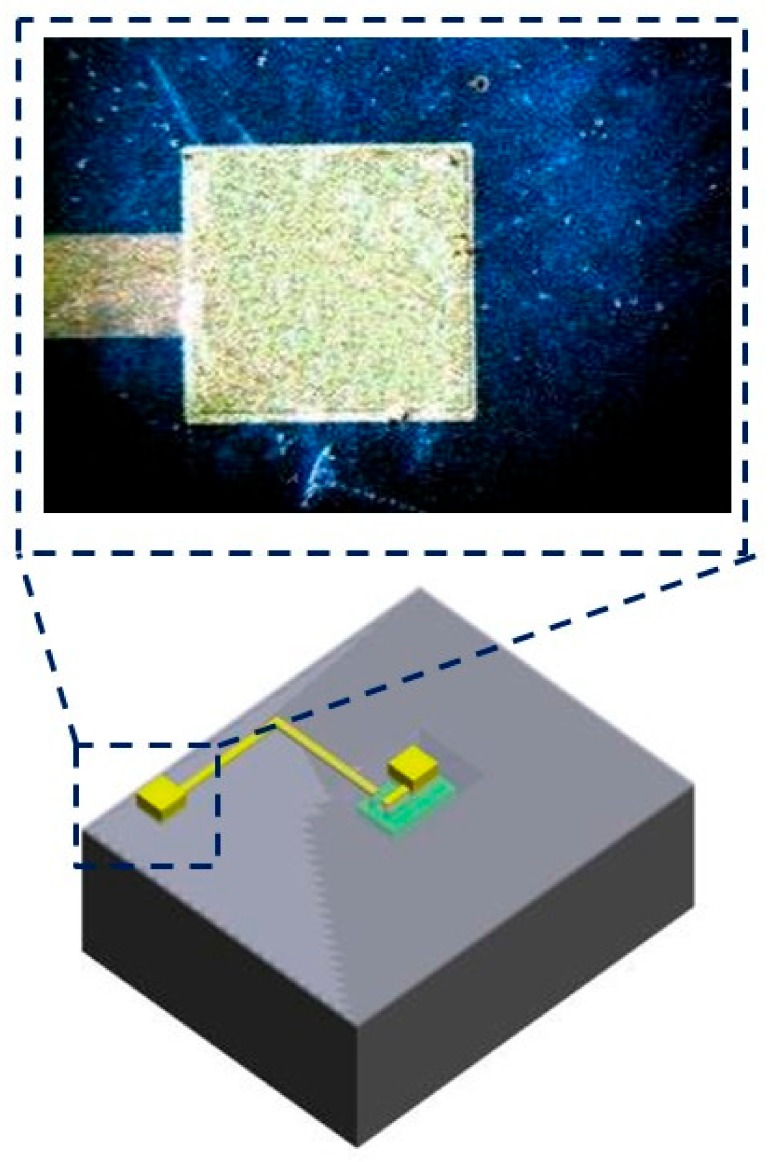
The close view of the second interconnection pad in step o. The second interconnection pad is 1500 μm × 1500 μm for Au coil pasting. There is a 60 μm of the overlap area can accommodate the misalignment.

Gold wet etching was used for the etchant solution and the second gold was patterned for the interconnection pad. Figure 5 shows the close look of the second interconnection pad in step o. The second interconnection pad is 1500 μm × 1500 μm for gold coil pasting.

Step p: Gold foil pasting

A gold coil of approximately 50 µm thickness was pasted on the interconnection pad by using hot embossing after the gold foil was patterned using the pulse laser.

Step q: Third parylene package

A third layer of parylene measuring 30 µm in thickness was deposited, and all elements were packaged.

Step r: Wafer removal

More than 60 μm thickness of parylene has adequate stiffness that the system can be peeled off directly from the silicon wafer and a flexible microsystem was obtained.

**Figure 6:**
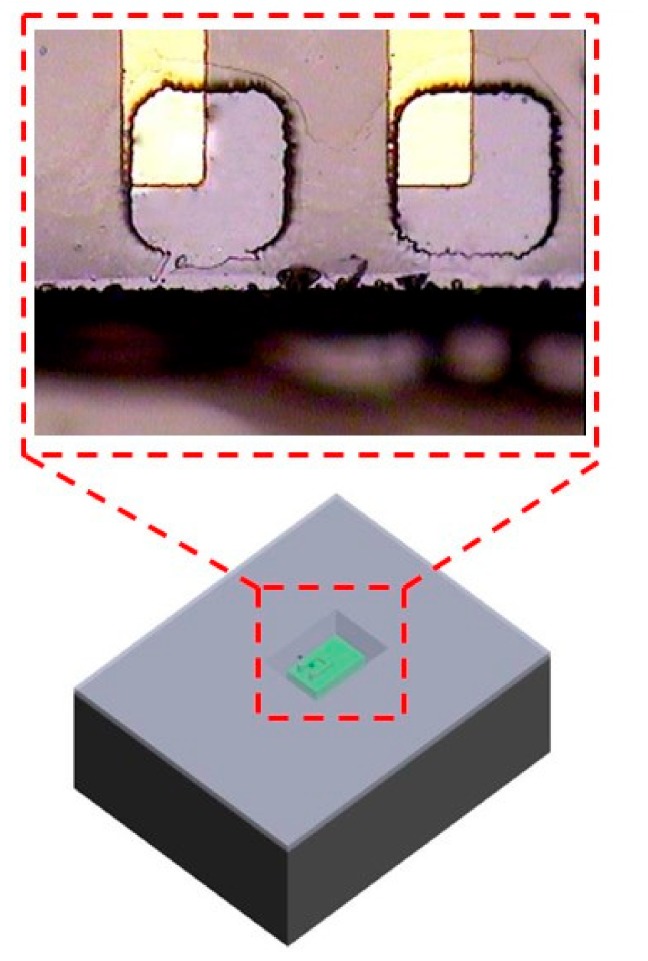
The patterns of via opening are larger than the pads of chip to accommodate the X and Y misalignments in step f.

Concerning the misalignments during the chip integration, X and Y axis misalignments of chip comes from the silicon trench etching and chip dicing. To overcome the misalignments, the patterns of via opening are larger than the pads of chip to accommodate the X and Y misalignments in step f shown in Figure 6. The Z axis of misalignment comes from the deviation of etching depth, and parylene has the excellent conformal deposition and trench filling capability, the discrepancy between IC and substrate can be larger than 2 μm. However, the conformal deposition capability of photoresist is poor than parylene, the discrepancy capability is limited by photoresist. 5 μm of AZ4620 was spun on the parylene, so the discrepancy (Z-axis misalignment) between IC and parylene is less than 5 μm.

## 4. Measurement Setup

**Figure 7:**
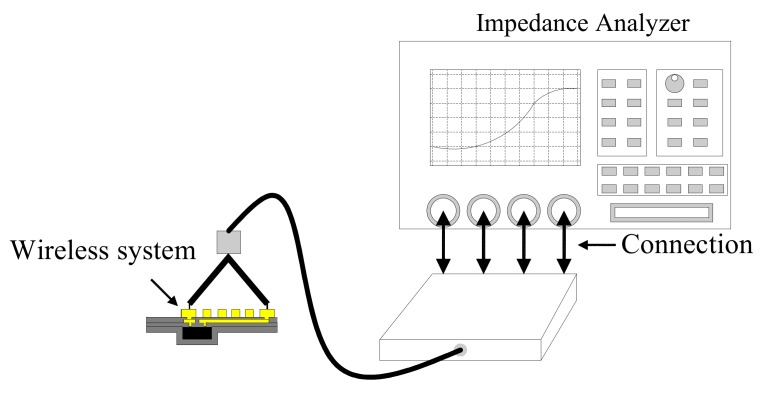
Inductance and resistance measurements.

When the coil was pasted on the system and the interconnection was completed before the uppermost parylene layer was covered, an Agilent 4294A impedance analyzer was used along with an Agilent 166334A probe to measure the inductance and resistance. Figure 7 illustrates the measurement setup involving the use of a probe to measure the pads of coils. The inductance and resistance of the system were acquired, and the Q-factor was calculated using (1).

## 5. Results and Discussion

### 5.1. Patterned Coil

**Figure 8:**
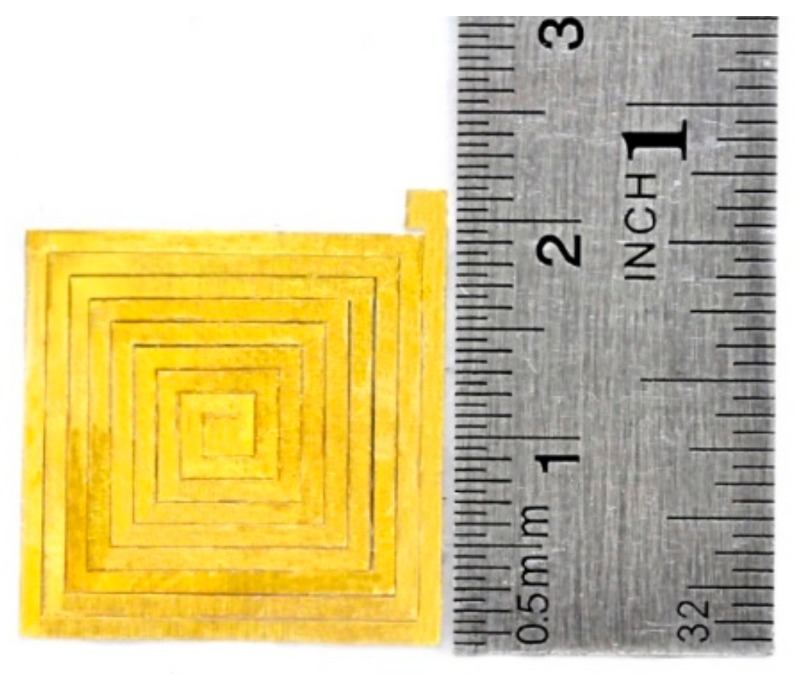
Laser-micromachined coil fabricated through laser cutting.

A gold coil of 50 μm thickness was created using a pulsed layer [24]. The coil specifications are shown in Table 1, and Figure 8 presents the machined coil produced using laser cutting.

### 5.2. Wireless Microsystem

**Figure 9:**
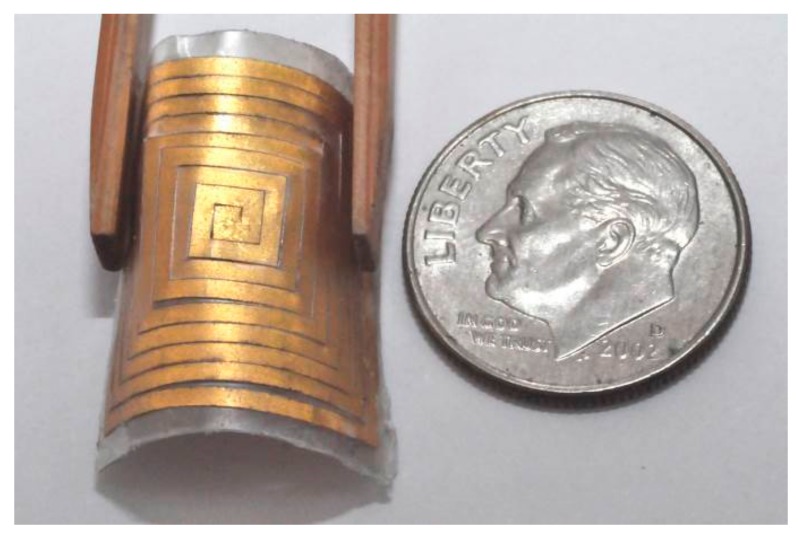
Front view of flexible wireless biomedical system.

**Figure 10:**
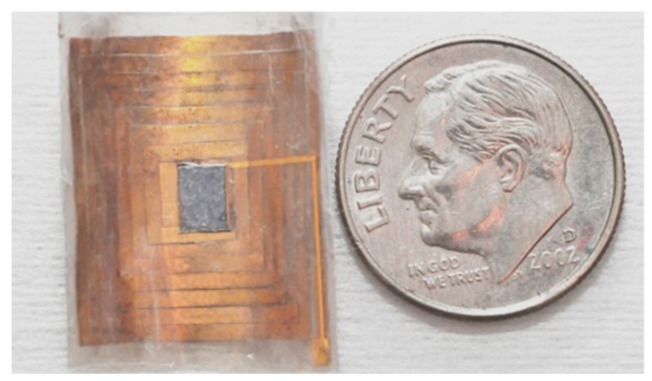
Back view of flexible wireless biomedical system.

The wireless microsystem was finished using the proposed process shown in Figure 4. Figure 9 and Figure 10 display the front and back views of the flexible wireless system, respectively. Table 2 shows the measurement results for the coil and the system at the operating frequency of 1 MHz. The Q-factor of the system was approximately 4.27.

## 6. Conclusions

This study presents a new 3D bottom-up packaging technology, which reduces the chip area without degrading performance. Parylene was used in this study as a flexible substrate for bottom-up embedding of a chip, insulation layer, interconnection, and inductors to create a flexible wireless biomedical microsystem. Because parylene and gold are bio-implantable materials of USP-VI grade, the entire wireless microsystem can be implanted on or inside the human body. A dummy chip was developed, and the measurement results indicate that at an operating frequency of 1 MHz, the Q-factor of the system was approximately 4.27.

## Figures and Tables

**Table 1:** Specifications of coil design.

Item	Specification
Material	Gold
Width of strip	1000 µm
Outer diameter	20 mm
The distance between strips of coil	~60 µm
Thickness	50 µm
Number of turns	8.5

**Table 2:** Measurement results for the coil and the system at an operating frequency of 1 MHz.

Item	Inductance (nH)	Resistance (mΩ)	Q-Factor
Induction coil	600	570.24	6.61
Induction coil with system	557	819.15	4.27
